# EU-US Relations: Reinventing the Transatlantic Agenda

**DOI:** 10.1007/s10272-021-0943-3

**Published:** 2021-01-26

**Authors:** Steven Blockmans

**Affiliations:** Director of Research, Centre for European Policy Studies, Place du Congrès 1, 1000 Brussels, Belgium

## Abstract

With Joe Biden’s victory, there is at least a four-year window to revive ‘an alliance of democracies’, face up to authoritarian powers and closed economies that exploit the openness on which American and European societies are built, and shape those parts of multilateralism that serve transatlantic interests.

## Damaged...

President Donald Trump's unabashed unilateralism has hurt EU-US relations. He has called the European Union a "foe" and "worse than China, just smaller" (Kwong, 2018). He celebrated Brexit and has encouraged other member states to leave the bloc. He has bullied democratic leaders such as Angela Merkel and embraced autocrats like Viktor Orbán. The latter has not helped the EU institutions in their search for supranational mechanisms to enforce compliance with rule of law conditions for membership.

Not only did the 45th President of the United States refuse to re-engage with the transatlantic trade and investment partnership (TTIP) agenda, which Barack Obama abandoned, but he also imposed "national security" tariffs on steel and aluminium imports from European allies and threatened that more might follow.[1] He also subjected European businesses to American extraterritorial jurisdiction more enthusiastically than any of his predecessors, in particular over his withdrawal of the US from the Iran nuclear deal (see Stoll et al., 2020).

Trump's retreat from the Paris climate deal, the Intermediate-Range Nuclear Forces treaty, the Open Skies agreement, and the World Health Organization (WHO) as well as his attacks against the WTO appellate body have rocked many Europeans' belief that they share common ground with their most important ally. In fact, Trump has been disdainful of European priorities, from climate change and efforts to improve global health, to human rights and development assistance.

As a result, US relations with the EU have become largely dysfunctional, and this comes at a time when unprecedented global health, economic and security challenges demand robust transatlantic leadership.

To be sure, transatlantic disarray is not solely due to Trump. After more than a decade of crisis management, the EU has seemed as likely to fall apart as to come together over the COVID-19 pandemic. The coronavirus crisis has ravaged societies and economies. Whereas EU member states reached a political agreement on a historic recovery package and a seven-year financial framework, those debates have also revealed ongoing differences on rule of law conditionality in the disbursement of funding that could widen once the worst of the pandemic is over.

## ...but not beyond repair

A second term for Trump would have almost certainly meant a further erosion of US democracy and the postwar liberal order. The EU would no longer have been able to put off facing the consequences of having an illiberal, anti-trade partner across the pond.

With Joe Biden's victory, there is at least a four-year window to revive 'an alliance of democracies', face up to authoritarian powers and closed economies that exploit the openness on which American and European societies are built, and shape those parts of multilateralism that serve transatlantic interests.

During the campaign, candidate Biden emphasised his long-standing belief that "Europe is the cornerstone of our engagement with the rest of the world and is the catalyst for our global cooperation".[2] As a passionate transatlanticist and multilateralist, Biden will instinctively turn to the EU as America's indispensable partner of first resort when it comes to addressing internation-

1 Clarification can be expected from the various WTO panel reports on complaints against the US tariff measures on steel and aluminium, which are due to be circulated soon. This includes complaints by China – DS 544; India – DS 547; the EU – DS 548; Canada – DS 550; Mexico – DS 551; Norway – DS 552; the Russian Federation – DS 554; Switzerland – DS 556 and Turkey – DS 564.

**Steven Blockmans**, Centre for European Policy Studies, Brussels, Belgium; and University of Amsterdam, Netherlands.

2 This resonated with the Remarks by Vice President Joe Biden to the Munich Security Conference (see United States Office of the Vice President, 2013).

al challenges, even if that partner has already made it clear to the incoming administration that it will not be dictated by the United States:

> The EU and the US should pursue common interests and leverage our collective strength to deliver results on our strategic priorities. We should always look for solutions that respect our common values of fairness, openness and competition – including where there are bilateral differences. (European Commission, 2020, 2)

## America, heal thyself before you attend to others

The 46th President's most immediate challenge will most likely not be abroad but an unenviable confluence of crises at home: COVID-19 vaccination management, post-pandemic economic recovery and deep social tensions. As the 6 January storming of the Capitol building by a mob of Trump supporters so brutally illustrated, Joe Biden will also have to contend with a much stronger radical conservative opposition than Barack Obama ever did.

Despite the many doubts sown about the American election process by Donald Trump and the legal challenges that remain, US democracy has survived its experiment with proto-fascism and will be strengthened in the next four years. This will be a boon for democratic forces around the world, especially in Europe. Recent developments in certain EU member states have shown that democratically elected leaders will try to use majoritarian rule to curb freedoms, overstep the constitutional limits of their powers, protect the interests of their cronies, and recycle themselves through seemingly free and fair elections. A Biden presidency is expected to strike up alliances that will solidify America's international role and put pressure on the illiberal and undemocratic leadership of third countries. This is good news for the EU and its drive to stop the corrosive effect of authoritarian tendencies within the bloc and strengthen rule of law mechanisms at the supranational level.

America's partners should therefore not be surprised, and should in fact welcome the likelihood that Biden's initial focus will necessarily be on domestic challenges. After all, the US is unlikely to be the type of consistent, outward-looking partner that Europeans need and want if it does not beat COVID-19, generate economic growth and work to heal its deep domestic divisions. And even if the Democratic Party holds a majority in both houses of Congress, the domestic forces that the Biden administration will have to contend with are likely to slow down the implementation of his ambitious foreign policy agenda.

## Reinvent transatlantic relations

While the era of American exceptionalism may be over,[3] a Biden Presidency will help to restore a balance of power and could help to reboot multilateralism. But even if the US rejoins the WHO, the Paris climate accords and the Iran nuclear deal, and works to strengthen the WTO, Biden's foreign policy will be more assertive and transactional in response to popular domestic demand. Europeans should not kid themselves into believing that transatlantic relations will return to the status quo ante. In all but name, the rallying cry of "America First" is here to stay. As a presidential candidate, Biden has vowed to prioritise investment in US green energy, childcare, education and infrastructure over any new trade deals. He has also called for expanded "Buy American" provisions in federal procurement, which has long been an irritant in trade relations with the EU. The EU will likely be forced to muster all the political will and resources at its disposal to carve a third way between the US and China, an issue which enjoys strong bipartisan support in Washington.

A new transatlantic agenda will demand more, not less, of Europe. The European Commission and the EU's High Representative for foreign affairs and security policy have understood this. In a call on the US to seize a "once-in-a-generation" opportunity to forge a new global alliance, they have made a detailed pitch to bury the hatchet on the sources of tension from the Trump era and meet the "strategic challenge" posed by China (European Commission, 2020, 1, 8). The idea is to revitalise the transatlantic partnership by cooperating on everything, from fighting cybercrime and shaping the digital regulatory environment, to screening sensitive foreign investments and fighting deforestation. An EU-US Summit in the first half of 2021 could be the moment to launch the new transatlantic agenda.

## Dealing with China

The new EU-US Dialogue on China is expected to provide a key mechanism for advancing shared transatlantic interests and managing differences on the best way forward. Topics include biomedical research, a green trade agenda, and – more acutely related to the system-

3 Richard Haass, former Director of Policy Planning for the United States Department of State and a close advisor to Secretary of State Colin Powell in the administration of Republican President Bush Jr., tweeted that "If the post-American era has a start date, it is almost certainly today", i.e. 6 January 2021.

ic rivalry with China – securing 5G infrastructure across the globe, opening a dialogue on 6G, widening cooperation on digital supply chain security through objective risk-based assessments, cybersecurity, free data flow on the basis of high standards and safeguards, cooperation on artificial intelligence, and fair taxation in the digital economy.

There is a genuine willingness in Europe to work with the US on the strategic challenges posed by China, but not at all costs. The provisional conclusion of talks on the Comprehensive Agreement on Investment (CAI) ahead of Biden's inauguration shows that the EU, led by Germany and France, is bent on protecting its commercial interests and will not slavishly follow a hegemonic US in decoupling from China. But by going soft on fundamental rights and enforcement mechanisms in the draft CAI,[4] in particular ILO standards on forced labour (cf. camps for Uighurs in Xinjiang province) and UN protected freedom of speech and assembly (in Hong Kong and elsewhere), the European collective has handed a victory to Beijing by splitting the aspired value-based transatlantic partnership. As a self-proclaimed "geopolitical" actor, the EU may have been shrewd in applying the realist approach of "principled pragmatism" before a Biden administration could affect some of its commercial interests,[5] but it still suffers from strategic myopia in defining relations in an increasingly bipolar world based on ideological lines (democracies vs authoritarian regimes). This episode places the new EU-US Dialogue on China on the back foot before it has even begun.

The news that, from the get-go, the Biden administration wants to sit down with its European allies to end the tug-of-war on trade is very welcome.[6] Resolving these and other issues with a commitment to improve the transatlantic level playing field is key to setting high standards, making critical supply chains more resilient and addressing China's unfair trade practices. And while the CAI is a meritorious attempt at getting Beijing to play by the rules, the EU would have stood stronger after consultation and in concert with the Biden administration.

## In conclusion

The greatest danger to a vital transatlantic bond will be Europe's temptation to believe that the relationship can go back to "business as usual". That would be a mistake. The EU-US alliance as we have known it is dead. A Biden administration will not want to "restore" the transatlantic partnership; it will want to reinvent it for a world full of economic, climate and health challenges, more diffuse power, rapid technological changes, greater insecurities and intensified global competition. Fortunately, this is well understood at EU headquarters and most of the member states capitals. But coming up with a common approach will hinge significantly on the two economies' ability to bridge existing divides over trade and technology policy. Using their combined influence, a transatlantic technology space could well form the backbone of a wider coalition of like-minded democracies.
